# Integrative Analysis of Transcriptomic and Epigenomic Data to Reveal Regulation Patterns for BMD Variation

**DOI:** 10.1371/journal.pone.0138524

**Published:** 2015-09-21

**Authors:** Ji-Gang Zhang, Li-Jun Tan, Chao Xu, Hao He, Qing Tian, Yu Zhou, Chuan Qiu, Xiang-Ding Chen, Hong-Wen Deng

**Affiliations:** 1 Center of Genomics and Bioinformatics, Tulane University, New Orleans, Louisiana, 70112, United States of America; 2 Department of Biostatistics and Bioinformatics, Tulane University, New Orleans, Louisiana, 70112, United States of America; 3 Laboratory of Molecular and Statistical Genetics, Hunan Normal University, Changsha, Hunan, 410081, China; University of Michigan, UNITED STATES

## Abstract

Integration of multiple profiling data and construction of functional gene networks may provide additional insights into the molecular mechanisms of complex diseases. Osteoporosis is a worldwide public health problem, but the complex gene-gene interactions, post-transcriptional modifications and regulation of functional networks are still unclear. To gain a comprehensive understanding of osteoporosis etiology, transcriptome gene expression microarray, epigenomic miRNA microarray and methylome sequencing were performed simultaneously in 5 high hip BMD (Bone Mineral Density) subjects and 5 low hip BMD subjects. SPIA (Signaling Pathway Impact Analysis) and PCST (Prize Collecting Steiner Tree) algorithm were used to perform pathway-enrichment analysis and construct the interaction networks. Through integrating the transcriptomic and epigenomic data, firstly we identified 3 genes (*FAM50A*, *ZNF473* and *TMEM55B*) and one miRNA (hsa-mir-4291) which showed the consistent association evidence from both gene expression and methylation data; secondly in network analysis we identified an interaction network module with 12 genes and 11 miRNAs including *AKT1*, *STAT3*, *STAT5A*, *FLT3*, hsa-mir-141 and hsa-mir-34a which have been associated with BMD in previous studies. This module revealed the crosstalk among miRNAs, mRNAs and DNA methylation and showed four potential regulatory patterns of gene expression to influence the BMD status. In conclusion, the integration of multiple layers of omics can yield in-depth results than analysis of individual omics data respectively. Integrative analysis from transcriptomics and epigenomic data improves our ability to identify causal genetic factors, and more importantly uncover functional regulation pattern of multi-omics for osteoporosis etiology.

## Introduction

Osteoporosis is a worldwide public health problem characterized by low bone mineral density (BMD) and a high risk of osteoporotic fracture [1]. BMD can be reliably and accurately measured and has high genetic determination with heritability of 0.5–0.9 [2], indicating that genetic factors play an important role in risk of osteoporosis. Recent advances in high-throughput technologies enable interrogation of various biological components on a genome wide scale (i.e., genomics, transcriptomics, epigenomics and proteomics), which have uncovered a number of risk factors/genes for human complex diseases and osteoporosis [3, 4]. However, these implicated genes explain no more than 10% of BMD variation in any individual human population [5]. The specific functional genomic/epigenomic variants and/or causal genes are largely unknown, and the molecular mechanisms by which the causal genes/variants function are even much less elucidated.

Identifying causal genes and characterizing their regulation patterns and functional contributions to osteoporosis risk is challenging because of its nature of complex genetic determination with a large number of genomic, transcriptomic, epigenomic, proteomic and environmental risk factors that often interact via biological networks [6]. So far, most of studies were focused on DNA, RNA, or protein levels individually and respectively, and rarely integrated evidences from multiple molecule levels to ascertain the importance of certain gene(s) for bone phenotypes. It is well known that genetic information is transcribed from DNA to mRNA, and then translated into proteins. Epigenetic factors (Epigenes) and their interactions (such as those between DNA methylation and miRNA) modulated by environment, affect gene expression (e.g., GXE interactions) into mRNAs/proteins and mRNA stability. Defects at any level (DNA, mRNA, epigenes and proteins) may or may not translate into the next level(s), to ultimately impact disease risk. While individual omics studies (genome/transcriptome/epigenome/proteome) are useful in identifying association of molecules with disease risk, they fall short of illuminating the underlying functional mechanisms. Therefore, it is usually not feasible for uni-omics studies to provide a comprehensive view of the genetic factors and their functions in the form of complex function/regulatory networks for the etiology of osteoporosis [7]. On the contrary, integrating multi-omics data may yield most thorough information to most powerfully and comprehensively identify molecular and genomic factors/mechanisms underlying the pathogenesis of osteoporosis, by identifying and characterizing those individual molecules as well as their involved complex regulation networks embedded in and across multi-level and multi-facet-omics data [8].

PBMs (peripheral blood monocytes) are established and accepted as a well working cell model for studying gene/protein expression patterns and their modulation mechanisms in relation to osteoporosis risk *in vivo* in humans [9, 10]. PBMs may act as precursors of osteoclasts [11, 12]. PBMs can migrate to bone surface and differentiate into osteoclasts. Blockade of the migration can relieve bone loss in a murine osteoporosis model by limiting bone homing of osteoclast precursors and reducing the number of mature osteoclasts attached to the bone surface [13, 14]. Abnormalities in PBMs have been linked to various skeletal disorders/traits [15, 16]. PBMs produce cytokines important for osteoclast differentiation, activation, and apoptosis [17]. In addition, PBMs are the only relatively most homogeneous known bone-significant cells that can be isolated fresh *in vivo* in large quantities for human population level omics studies [18]. Therefore, we will use PBM for this first integrative multi-omics study in the bone field.

The present study represented our pursuant effort to ascertain the significance of the BMD-associated genes and their functional networks with integrative evidences from three omics data simultaneous from a same sample set. Therefore, this study is expected to pioneer an innovative approach to greatly and most comprehensively enhance our understanding of molecular genetic mechanism in osteoporosis. In this work, we developed an innovative analytic method for systemically integrating and analyzing multiple omics datasets and applied this approach in an integrative trans-omics study for BMD variation, for which we simultaneously interrogated transcriptomic and epigenomic profiles in the same set of biological samples and constructed a global model/pattern of (epi-) genome organization and gene regulation for BMD-associated genetic factors. Together, these analyses would provide a much more comprehensive system-level perspective on BMD than any individual uni-omics data analysis.

## Methods and Materials

### Subject Recruitment and Data Generation

#### Subjects

The study was approved by the Research Administration Department of Hunan Normal University. Five high hip BMD (mean±SD = 1.10±0.08 g/cm^2^) female subjects and five low hip BMD (mean±SD = 0.74±0.03 g/cm^2^) female subjects were recruited from Changsha City and its vicinity in the Mid-south area of China during 2010–2011. These subjects were selected from top one hundred and bottom one hundred hip BMD subjects among 1,915 healthy female subjects aged of 20 to 45 years. All subjects signed informed-consent documents before entering the project. For each subject, we collected information on age, sex, medical history, family history, menstrual history, smoking history, physical activity, alcohol use, tea and coffee consumption, diet habits, etc. Female subjects all had regular menses to eliminate the dramatic aging effects due to menopause on female BMD. Subjects with chronic diseases and conditions that potentially affect bone mass were excluded from the study. One hundred and fifty milliliters of peripheral blood were drawn for each selected subject. The characteristics of the subjects were shown in Table 1.

**Table 1:** Basic characteristics of study subjects.

**Trait**	**Low BMD**	**High BMD**
Age(years)	22.20±1.30	21.60±1.82
Height(cm)	160.60±2.51	159.20±5.08
Weight(kg)	55.00±8.97	61.90±5.44
Hip BMD(g/cm^2^)	0.74±0.03	1.10±0.08
Spine BMD(g/cm^2^)	0.83±0.07	1.06±0.16

Note: BMD, bone mineral density.

#### BMD measurement

BMD (g/cm^2^) at the lumbar spine (L1–4, anteroposterior view) and the hip, including femoral neck (FN), trochanter and intertrochanteric regions, were measured using the Hologic QDR 4500 W bone densitometer (Hologic, Waltham, MA, USA). The total hip BMD was a combined value at the three measured regions. The densitometer was calibrated daily, and long-term precision was monitored with the control vertebral phantom. The coefficient of variation (CV) of measured total hip BMD values was 1.34%.

#### Monocyte isolation

A monocyte negative isolation kit (Order No. 130-091-153, Miltenyi Biotec Inc., Auburn, CA, USA) was used to isolate circulating monocytes from 150mL whole blood following the procedures recommended by the manufacturer. The kit contains Cocktail of biotin-conjugated monoclonal antibodies against CD3, CD7, CD16, CD19, CD56, CD123 and Glycophorin A to deplete Non-monocytes, i.e. T cells, NK cells, B cells, dendritic cells and basophils, leaving monocytes untouched, pure, viable, and free of the surface-bound antibody and beads. The purity of the isolated monocyte was monitored by flow cytometry (BD Biosciences, San Jose, CA, USA) with fluorescence labeled antibodies PE-CD14 and FITC-CD45 (S1 Fig), and determined to be 84.5% in our samples.

### DNA and total RNA extraction

Genomic DNA was extracted from the freshly isolated PBMs. The concentration of DNA was assessed by using Qubit 2.0 Fluorometer (Invitrogen,). Total RNA from monocytes was extracted using a Qiagen RNeasy Mini Kit (Qiagen, Inc., Valencia, CA, USA). RNA integrity was assessed by using an Agilent 2100 Bioanalyzer (Agilent Technologies, Palo Alto, CA, USA).

### Gene Expression Arrays

Affymetrix GeneChip Human Exon 1.0 ST Array (Affymetrix, Santa Clara, CA) was used to evaluate genome-wide gene expression levels. This exon array contains ~1.4 million probesets consisting of ~5.4 million probes and profiles over 17,000 well-annotated gene transcripts in the human genome. The experiment was performed by Capitalbio Cor. (Beijing, China), according to the protocol provided by the array supplier. Double-stranded cDNA was synthesized by using the Superscript Choice System, followed by an *in vitro* transcription reaction with a T-7 (dT24) primer to produce biotinylated cRNA. The full-length cRNAs were fragmented to 20 to 200 bp and hybridized to Affymetrix GeneChip Human Exon 1.0 ST Array. 100 ng of total RNA was amplified and labeled using the Affymetrix Whole-Transcript (WT) Sense Target Labeling Protocol without rRNA reduction. Affymetrix GeneChip Human Exon 1.0 ST arrays were hybridized with 11 μg of labeled sense DNA, washed, stained, and scanned according to the protocol described in WT Sense Target Labeling Assay Manual (Version 4; FS450_0007). All the expression data of the 10 samples had been preprocessed using robust multichip average (RMA) normalization.

### miRNA microarray

miRNA microarray was performed by Capitalbio Cor. (Beijing, China). Briefly, 1 μg of total RNA from each sample was end-tailed with Poly(A) and ligated to the biotinylated signal molecule (FlashTag™ Biotin RNA Labeling Kit). Hybridization (Affymetrix Genechip Hybridization Oven 640) was carried out on Affymetrix genechip® miRNA 2.0, which contains 15,644 mature miRNA probes from miRBase V15 (all 131 organisms), 2,334 snoRNAs and scaRNAs and 2,202 probe sets unique to pre-miRNA hairpin sequences. The hybridized chips were washed (Affymetrix Fluidics Station 450), stained with phycoerythrin-streptavidin, and scanned using an Affymetrix GeneChip® Scanner 3000 7G. Scanned images were quantified by GeneChip® Command Console® Software. miRNA QC Tool software (www.affymetrix.com/products_services/arrays/specific/mi_rna.affx#1_4) was used for data summarization, normalization, and quality control.

### MeDIP-seq

MeDIP-seq was performed by BGI Inc (Shenzhen, China). Briefly, genomic DNAs were sonicated to ~100-500bp with a Bioruptor® NGS Sonication (Diagenode). 1.5μg of sonicated DNA was end-repaired, 3’A added, and ligated to single-end adapters following the standard Illumina genomic DNA protocol. The double-stranded DNA was denatured with 0.1 M NaOH to generate single-stranded DNA molecules. Methylated DNAs were enriched by immunoprecipitation with an anti-5-methylcytosine monoclonal antibody. Q-PCR was used to confirm the enrichment of methylated region. Precipitated DNA fragments were then purified and amplified. The PCR products were separated on 2% agarose gel to select fragments in the ~220-320bp size range. The completed libraries were quantified and checked for quantity with Agilent 2100 Bioanalyzer and ABI StepOnePlus Real-Time PCR System, and subsequently sequenced on Illumina HiSeq 2000 (with single-end, 49bp reads). Sequencing reads that passed through the HiSeq 2000 quality filter were aligned to the human genome reference sequence (GRCh37/hg19) using SOAP (Version 2.20). DNA methylation profiles were inferred from the uniquely aligned reads by using the MEDIPS analysis package [19]. In this study we focused on the methylation profiles in promoter regions of protein coding genes and miRNA gene regions, and the corresponding genomic locations were obtained from the UCSC Genome Brower.

All the data were submitted to the GEO repository, which can be accessed as the SuperSeries accession GSE62589 (http://www.ncbi.nlm.nih.gov/geo/query/acc.cgi?acc=GSE62589).

## Data Analysis

**Fig 1:**
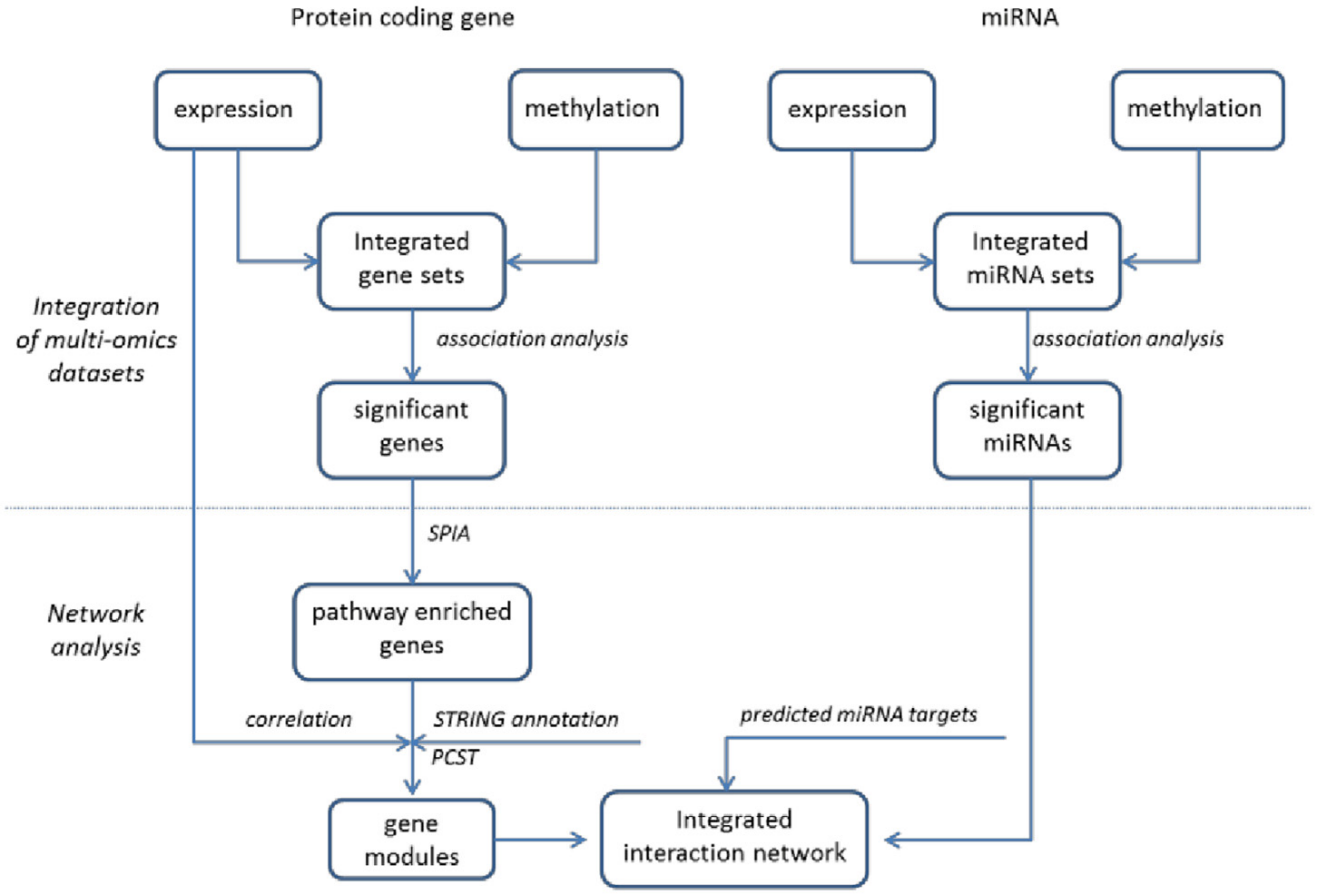
Sketch of the multi-omics data analysis workflow in this study.

We divided our analysis into two parts as shown in Fig 1: 1) identify BMD-associated protein coding genes and miRNAs by integrating transcriptomic and epigenomic data; 2) subsequently discover networks implicated in BMD through network analysis with transcriptomic and epigenomic data.

### Integration of multi-omics datasets

To synthetize the information obtained from expression and methylation profiles for each protein coding gene (or miRNA), we developed an algorithm to compute crosstalk scores, which combine the disease-association evidence from individual uni-omics studies through a meta-analysis based approach [20].

First, we determined gene-to-phenotype association scores separately for individual uni-omics data. For gene *g*, compute scores $S_{g}^{k}(X_{k},\;Y)$ (*k* = 1,…, *D*, where D is the number of omics data), capturing the relationship between different genomic measurements *X*_*k*_ (e.g., RNA transcriptomics and epigenomics) and a phenotype *Y*. In this study $S_{g}^{k}(X_{k},\;Y)$ was a t-test statistic that measures the differentiation between high and low BMD groups for *k*-th omics data. In each omics data, the most significant probe was chosen to represent the gene if more than one probe are mapped to the given gene; Second, integrated the gene-to-phenotype association scores ($S_{g}^{1}(X_{1},\;Y),\;{\ldots ,S}_{g}^{D}(X_{D},\;Y)$) into a gene-specific score ($S_{g\_meta}(S_{g}^{1},\;S_{g,\;\ldots ,\;}^{2}S_{g}^{k})$) by a meta-analysis based approach [20]:

$$S_{g{\_}_{meta}}\left(S_{g}^{1},\;S_{g,\;\ldots ,}^{2}S_{g}^{k}\right)=\sum {}_{k=1}^{D}\left|Z_{g}^{k}\right|\;\;\;\;\left(k\in \left(1,\ldots ,\;D\right)\right)$$ (1)

$Z_{g}^{k}\;=\;\frac{S_{g}^{k}(X_{k},\;Y)}{sd(S_{g}^{k})}$ was the standardized score of gene g in the data set *k*, and $sd(S_{g}^{k})$ was derived by permutation under the null hypothesis that there is no association between gene *g* and *Y* in *k*-th omics data. When there is no association between gene *g* and phenotype *Y*, $\left|Z_{g}^{k}\right|$ follows a Half-normal distribution. For two omics data, the cumulative density function of *S*_*g_meta*_ is defined as:

$$F\left(s\right)=\iint {}_{-\infty }^{x+y\leq s}f\left(x,y\right)dxdy={\left[2\emptyset \left(\frac{\sqrt{2}}{2}s\right)-1\right]}^{2}$$ (2)

Finally, a *P*-value for the combined score was adjusted using false discovery rate (FDR) in order to correct for multiple hypothesis testing [21].

### Network analysis

To construct biological modules associated with BMD status, we developed a three-step methodology as following:

#### Step 1

Conducted a Signaling Pathway Impact Analysis (SPIA) to perform pathway-enrichment analysis [22]. We used the mRNA data of the set of genes with p-value≤ 0.05 in above mentioned integration analysis as the seed genes, which incorporated the information from gene expression data and methylation data for a given gene. In SPIA, two statistical measurements, *P*_*NDE*_ (probability of differential expressed genes) and *P*_*PERT*_ (probability of perturbation) were calculated. *P*_*NDE*_ and *P*_*PERT*_ measure the overrepresentation of the differentially expressed genes in a pathway and the abnormal perturbation of a specific pathway, respectively. *P*_*NDE*_ and *P*_*PERT*_ are finally combined in one global probability value, *P*_*G*_, which calculates the significantly enriched pathway according to both the over-representation and perturbation-based evidence. The pathway information used in the present study was obtained from the Kyoto Encyclopedia of Genes and Genomes (KEGG, http://www.genome.jp/kegg/) database.

#### Step 2

The procedure of the network reconstruction was as followed:

1. Chose the genes of those significant pathways identified by SPIA. We assigned weights to both nodes (V) and edges (E). Node weights corresponded to the combined score (*S*) of protein coding genes derived from integration analysis and the edge weights corresponded to the confidence on that interaction between these genes. The edge weights were derived based on two kinds of evidence: interaction score for each edge from the Search Tool for the Retrieval of Interacting Genes (STRING) database and correlation (Pearson correlation) between nodes in mRNA data. Finally we combined them into a single score using a naive Bayesian approach to measure the interaction evidence among nodes [23].
2. Used PCST (Prize Collecting Steiner Tree) algorithm to construct the interaction networks for the chosen protein coding genes. Given a *G = (V*, *E)*, PCST is to find a connected sub-network *G*′ = (*V*, *E*) that minimizes the following function:

$$G={\text{min}}_{\begin{array}{l} \;E^{\prime }\subseteq E;\;V^{\prime }\subseteq V\\ (E^{\prime },\;V^{\prime })connected \end{array}}\left({\sum^{}}_{e\in E\prime }\;c_{e}-\lambda {\sum^{}}_{i\in V\prime \;}b_{i}\right)$$ (3)

The node prize *b*_*i*_ was computed by *b*_*i*_ = −log *p*_*i*_, where *p*_*i*_ was the p-value of node *i* in integration analysis. And the costs of edges *c*_*e*_ was $c_{e}\;=\;1-\prod_{j}^{k}R_{j}$ with *R*_*j*_ for the string score for the edge’s interaction evidence. The parameter λ regulates the trade-off between the cost of new edges and the prize gained by bringing in a new gene, and its value indirectly controls the size of the final sub-networks *G*′. In this analysis, all results presented here were obtained with *λ* = 0.62. In order to choose *λ*, we solved the PCST problem, varying *λ* between 0.01–1.00 in increments of 0.01, and choose the value of *λ* at which 70% of the essential nodes of simulated network of similar size were recovered.

#### Step 3

Generated miRNA-mRNA correlation networks. One major function of miRNAs is the cleavage of transcripts of its target genes at the post-transcriptional level. Thus, we were most interested in a negative correlation between miRNA and gene expression. Thus we built the miRNA-mRNA network via the following three steps: First, the Pearson’s correlation was used to detect correlations between the expression profiles of miRNAs (p-value less than 0.05 in integration analysis) and protein coding genes within the network identified in step 2. The cut-off for the correlation coefficient was set as -0.55. Second, screened the miRNA-mRNA pairs identified above by Exiqon miRSearch, TargetScan and microRNA.org, and identified the miRNA-mRNA pairs with miRNA–target relationships. Third, constructed miRNA-mRNA networks based on their correlations and miRNA–target relationships.

## Results

### Identification of BMD-associated genetic factors by integration analyses

We integrated transcriptomic and epigenomic data to identify the miRNAs and protein coding genes associated with BMD status. Table 2 demonstrated the protein coding genes and miRNAs that showed significant associations with BMD status. At false discovery rate (FDR) ≤ 0.05, 9 genes were identified. To validate the biological importance of 9 genes, the large GWAS meta-analyses of GEFOS2 (Genetic Factors for Osteoporosis Consortium) was used to perform *in silico* validation association. GEFOS-2 is the largest meta-analysis to date in the bone field, including 17 GWASs and 32,961 individuals of European and East Asian ancestry [24] (http://www.gefos.org/?q=content/data-release). It was shown that rs3747486 and rs3812999 in *RNF40* were associated with FN BMD in GEFOS2. Gene *ALDOA* also was found to be associated with FN BMD in GEFOS2, with *P* = 2.0E-2.

**Table 2:** 9 protein coding genes and 2 miRNAs identified in integration analysis.

Gene ID	*P*_exp	FDR_exp	*P*_methy	FDR_methy	*P*_inte	FDR_inte
*RNF40*	4.75E-4	0.83	0.11	0.65	1.53E-8	3.32E-4
*FAM50A*	6.18E-3	0.83	2.15E-3	0.65	7.55E-7	5.46E-3
*ZNF473*	5.97E-3	0.83	2.17E-3	0.65	6.75E-7	5.46E-3
*PDXP*	6.64E-4	0.83	0.18	0.65	3.79E-6	0.02
*CYP2E1*	0.44	0.83	1.96E-4	0.65	6.46E-6	0.02
*TMSB10*	0.77	0.84	4.44E-5	0.65	1.16E-5	0.03
*TMEM55B*	3.90E-3	0.83	2.86E-2	0.65	1.03E-5	0.03
*SH3BP1*	6.64E-4	0.83	0.33	0.65	1.19E-5	0.03
*ALDOA*	0.32	0.83	3.55E-4	0.65	2.27E-5	0.05
**miRNA ID**						
hsa-mir-4291	0.04	0.95	0.01	0.22	5.57E-4	0.05
hsa-mir-1253	0.72	0.98	5.99E-4	0.10	7.06E-4	0.05

Notes: *P*_exp is *P*-value of expression data; FDR_exp is FDR-value of expression data; *P* _methy is *P*-value of methylation data; FDR_methy is FDR-value of methylation data; *P* _inte is *P*-value of integration analysis; FDR_inte is FDR-value of integration analysis.

We also screened each uni-omics data using t-test and there was no gene that showed significant association with BMD at FDR≤ 0.05. Most gene captured by integration analysis showed stronger evidences of differential expression and methylation levels between high and low BMD groups. For example, the significance level of *FAM50A* was 6.18E-3 and 2.15E-3 in gene expression data and DNA methylation data, respectively. Integration analysis with gene expression and DNA methylation data generated a p-value of 7.55E-7, which was still significant after multiple-testing correction (FDR = 5.46E-3). Pearson correlation coefficient between expression and methylation of gene *FAM50A* showed -0.85 implying that promoter’s hypomethylation were associated with increased expression of genes. Similarly, in integration analysis genes TMEM55B and *ZNF473* (*P* = 1.03E-5 and 6.75E-7 respectively) showed significant consistent association with BMD at both gene expression level and methylation levels with negative correlations between gene expression and promoters methylation (*ρ* = -0.73 and -0.85 respectively).

Additionally, we performed the integration analysis of miRNAs expression and methylation data. In our data, there are 168 miRNAs with both expression and methylation data. After the multiple-testing correction, two miRNAs (hsa-mir-4291 and hsa-mir-1253) were identified to be significantly associated with BMD status with FDR≤ 0.05.

### Network analysis identified one BMD-related module

In the first step, 139 KEGG (Kyoto Encyclopedia of Genes and Genomes) pathways were available for the gene set enrichment analysis and the most significant pathways ranked by SPIA (Signaling Pathway Impact Analysis) were shown in Table 3. When considering both over-representation evidence and perturbation evidence with FDR≤ 0.20, we identified one significant pathway, acute myeloid leukemia, with FDR = 6.25E-2. It can be observed that the significant evidence of this pathway was mainly due to the strong evidence of over-representation (*P*_NDE_ (*P* value of over-representation evidence) = 1.40E-3). Only considering the over-representation evidence, we identified another two significant pathways with FDR≤0.20, Insulin signaling pathway and mTOR (mammalian target of rapamycin) signaling pathway, which has been reported to be associated with osteoporosis [25–27]. In this study no pathways with significant perturbation evidence was identified with FDR≤0.20.

**Table 3:** Top three pathways identified by SPIA.

Pathway	*P*_NDE_	FDR_NDE_	*P*_PERT_	FDR_PERT_	*P*_G_	FDR_G_
**Acute myeloid leukemia**	1.40E-3	0.11	0.11	1.00	1.54E-3	6.25E-2
**Insulin signaling pathway**	6.09E-3	0.19	0.79	1.00	3.05E-2	0.55
**mTOR signaling pathway**	7.11E-3	0.19	0.95	1.00	4.06E-2	0.55

Notes: SPIA: Signaling Pathway Impact Analysis. *P*_NDE_ is the *P* value of over-representation evidence, *P*_PERT_ is the *P* value of perturbation evidenceand P_G_ is the P value of combined over-representation evidence and perturbation evidence.

**Fig 2:**
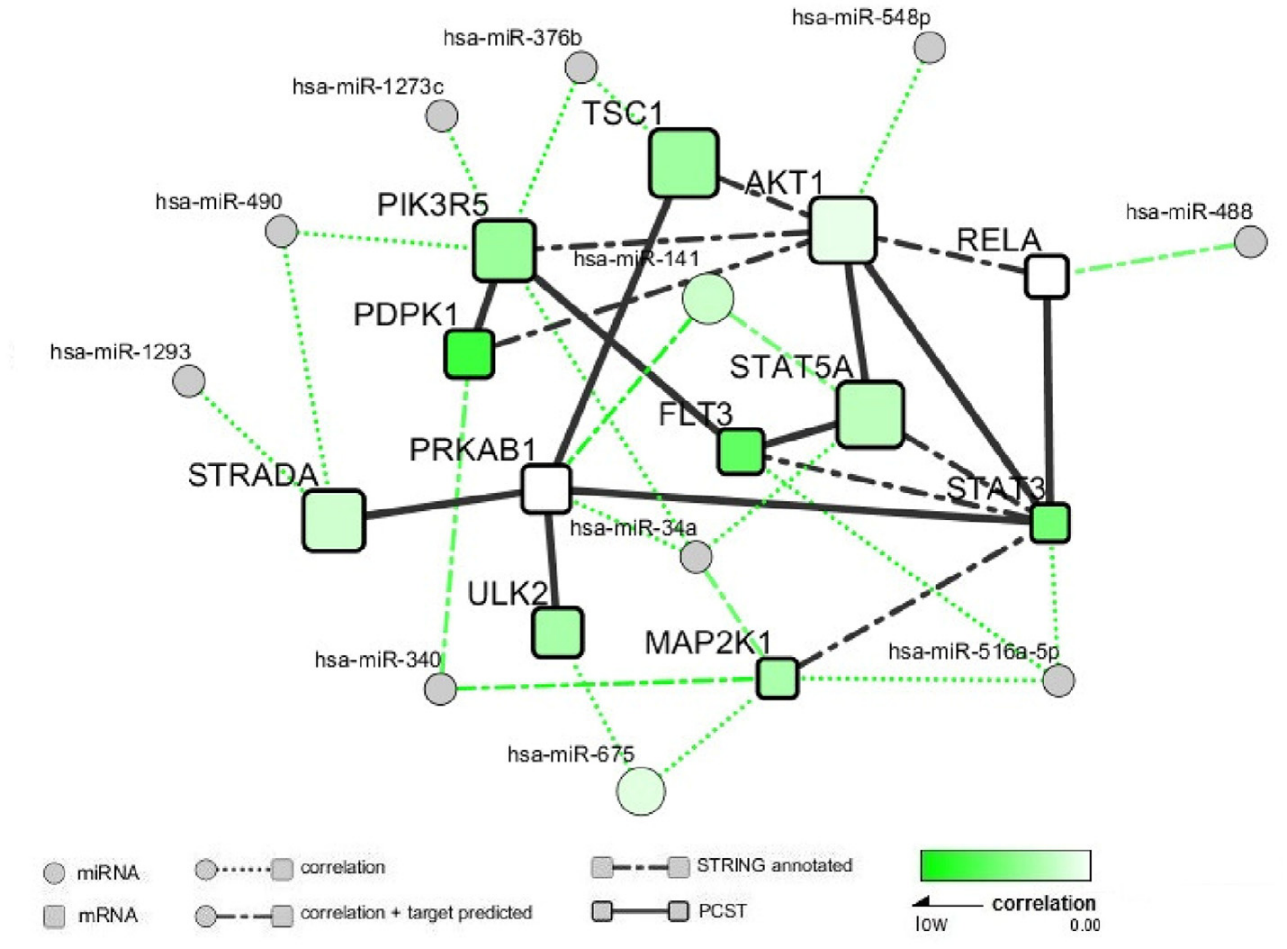
The interaction module inferred in network analysis. Genes were represented by squares and connected each other with solid lines. miRNAs were represented by circles. Node size was proportional to the absolute value of the combined *S* score of integration analysis. Node color represented the strength of negative correlation between gene expression profile and DNA methylation level. The direct gene interactions using dot-dash lines between genes based on annotation from STRING database. Abbreviations: PCST (Prize Collecting Steiner Tree); STRING (Search Tool for the Retrieval of Interacting Genes).

In the second step, we built a protein coding gene interaction network (G) with PCST (Prize Collecting Steiner Tree). We used the significant genes with FDR≤ 0.05 in the integration analysis and those belonged to those three pathways identified by SPIA and with *P*-value≤ 0.05 in the integration analysis. In total 28 genes were selected for the gene interaction network analysis. We identified one network module with 12 genes as shown in Fig 2. The gene interactions observed among these 12 genes suggested that there exits the function relevance among these genes. Some genes in this module have been known to be associated with BMD or be overlapped with BMD QTL regions, such as *PIK3R5*, *STAT5A* and *AKT1* [28–31]. Interestingly, most of the genes among these 12 genes showed consistent changes in gene expression and DNA methylation levels across high and low BMD groups and strong negative correlations were observed between DNA methylation status and expression of these genes. As an example, gene *PIK3R5* showed consistent association on gene expression level (*P* = 0.01) and DNA methylation level (*P* = 0.09), and a strong negative correlation was observed between DNA methylation status and expression of *STAT5A*, *FLT3*, *PDPK1* and *TSC1*.

In the third step we incorporated the miRNAs to the protein coding gene network to explore the regulation pattern of gene expression by miRNAs. We divided the miRNAs into two parts: the miRNAs with both expression and methylation data (miRNA_EM_) and the miRNAs with only expression data (miRNA_E_). We selected the miRNAs with *P*≤ 0.05 from both parts respectively and obtained 38 and 21 miRNAs from miRNA_E_ and miRNA_EM_ respectively. The pair-wise gene expression correlations between the 59 miRNAs and 12 protein coding genes were calculated. Finally, we identified 11 miRNAs whose correlations with 12 protein coding genes were less than -0.55. The results of correlation analysis of miRNAs and protein coding gene expressions were shown in Fig 2 Among these 11 miRNAs, has-mir-141 and has-mir-675 have both expression and methylation data. It was noted that there were two pairs miRNA- target gene (hsa-mir-141- *PRKAB1*, hsa-mir-340-*MAP2K1*) relationship. It was observed that there was a negative correlation between expression and methylation data of hsa-mir-141, and hsa-mir-141 showed consistent change on gene expression level (*P* = 0.03) and DNA methylation level (*P* = 0.15). This may suggest that the difference of hsa-mir-141 methylation levels between high and low BMD groups may contribute the change of its expression levels between two groups. In addition, we found *PRKAB1* is a target gene of hsa-mir-141 and only expression level of *PRKAB1* showed the difference between the two BMD groups, while there was no correlation between expression and methylation data of *PRKAB1*. This result indicated that the expression change of *PRKAB1* may be regulated by hsa-mir-141 and not by its methylation level.

## Discussion

In the present study, we developed a novel analysis framework to integrate multi-omics data to comprehensively explore the casual genetic factors and functional networks associated with BMD using transcriptomic and epigenomic profiling.

In the integrative analysis, we identified 9 protein coding genes and 2 miRNAs that were associated with BMD. Furthermore, 3 genes identified in our integration analysis were also replicated at DNA level in the two largest GWAS meta-analyses in the field. The replicated findings across different omics data warranted the significance of these genes to hip BMD. Additionally our pathway analysis using SPIA identified three significant pathways, acute myeloid leukemia pathway, Insulin signaling pathway and mTOR signaling pathway, which are associated with BMD status. The mTOR signaling pathway integrates both intracellular and extracellular signals and serves as a central regulator of cell metabolism, growth, proliferation and survival. Previous evidence showed that mTOR signaling contributed to chondrocyte differentiation and long bone growth [32] and osteoclasts apoptosis [33]. Insulin and its downstream signaling pathway were indispensable for postnatal bone growth and turnover by having influence on both osteoblast and osteoclast development [34]. Insulin signaling in osteoblasts not only modulated bone growth and turnover but was also required for energy metabolism [35].

One challenge in integration analysis for multi-omics data is the exploration of the interactive connections among different genes/factors. In this study, by a network analysis, we identified a network module including the miRNAs, mRNAs and DNA methylation associated with BMD. In this module, four possible gene expression regulation patterns were observed: 1) Regulation by promoter methylation. One example is *PIK3R5*, which showed the association signals from both transcript and methylation levels, and there was a negative correlation between these two levels as expected. This observation indicated the change of methylation level in *PIK3R5* may contribute to its differential expression between high and low BMD groups; 2) Regulation by miRNAs. In the module, has-mir-141 and *PRKAB1* both showed differential expression, while their expression profiles displayed a strong negative correlation. With the existent knowledge that PRKAB1 is a target gene of has-mir-141, we can deduce that the differential expression of *PRKAB1* was partially due to the differential expression of hsa-mir-141. 3) Regulation by transcription factors. Gene *AKT1* showed a strong association with BMD status, but we didn’t detect significant association between its methylation level and BMD status. Meanwhile, *AKT1* isn’t a target gene of any miRNA with significant association with BMD status. Among the nodes connected with *AKT1*, *STAT5A* showed a strong positive correlation. Thus we can infer that *STAT5A* may involve the regulation of *AKT1* expression. In agreement with our findings, previous studies also proved *STAT5A* play an important regulation role in *AKT1* [36]; 4) Co-regulation by promoter methylation and miRNAs. *PDPK1* was upregulated and hypermethylated in its promotor in the low BMD group compared to the high BMD group. Meanwhile, hsa-mir-340, which was downregulated in low BMD group, correlated with the expression level of its target gene *PDPK1* with the Pearson correlation, -0.65. Hence, the expression of *PDPK1* gene may be co-regulated by its promotor methylation and hsa-mir-340. Gene co-regulation mediated by both miRNA and promoter methylation occur in the human genome [37].

Some genes/miRNAs in the network have been shown as important modulating factors for bone development or remodeling. For example, *STAT3* mutant mice exhibit decreased bone density, bone volume, and increased numbers of TRAP-positive osteoclast [38]. *JAK2/STAT3* signaling plays important roles in the receptor activator of nuclear factor-kappaB ligand (*RANKL*)-mediated osteoclastogenesis [39, 40]. *STAT5A* is a member of *STAT* genes, which are thought to play a major role in bone growth and biological responses [41]. *STAT5A* knockout mice showed obviously defective bone development [42]. Our result indicated that the expression of gene *STAT5A* may be regulated by their methylation levels, respectively. *AKT1* (v-akt murine thymoma viral oncogene homolog 1) is a protein coding gene which regulates many processes including proliferation, cell survival, growth and angiogenesis. Global deficiency of *AKT1* caused a reduction in BMD, femoral cortical thickness and volume and genetic *AKT1* deficiency diminished the rate of proliferation of osteoblast progenitors and impaired osteoclast differentiation in primary culture [43]. FMS-related tyrosine kinase 3 (*FLT3*) has been shown to play a critical role in the osteoclast differentiation and function [44]. *FLT3* polymorphisms play a role in determination of BMD and subsequent fractures in postmenopausal women [45]. In addition, miR-141 remarkably modulated the BMP-2-induced pre-osteoblast differentiation through the translational repression of Dlx5, which is a bone-generating transcription factor expressed in pre-osteoblast differentiation [46]. miR-34a and its target protein Jagged1 (*JAG1*), a ligand for Notch 1 play a role in osteoblastic differentiation [47]. In a recent study, Krzeszinski *et al*. reported that miR-34a overexpression transgenic mice exhibited lower bone resorption and higher bone mass; while miR-34a knockout and heterozygous mice exhibited elevated bone resorption and reduced bone mass [48].

Limitations of our study include the facts that: 1) the sample size of 10 subjects may appear to be a little bit small. However, due to the polygenic architecture of BMD, this trait arises in each individual from the combined effects of a large number of genetic variants [49]. In most cases, there is still enough statistical power to identify a part of the causal genetic variants even with small sample size. As a simple numerical example, one complex trait A is associated with 100 genes. With a sample size, for each gene there is very small power to be identified in an association analysis with trait A (e.g. 3%). However, we still can have high probity 95.24% to identify at least one gene (p = 1-(1–0.03)^100^) in the first screen. From this example, we can find out that it is due to the polygenic architecture of BMD that we can identify some genes and genetic factors associated with BMD even with small sample size and reduced power in a pilot screening study. However, if an independent endeavor is pursued with an aim to replicate the initial finding, the power would then be only 3%. Additionally, this current study is not a prevailing and traditional uni-omics study. The multi-omics study can provide more comprehensive and thorough information than uni-omics studies. The identified genes and pathways showed consistent or collectively appreciable effects on BMD variation and data from earlier studies unambiguously confirmed our results, which attested to the reliability and robustness of our results and multi-omics approach. Our integrative trans-omics analysis of multi-omics data thus leaded to a comprehensive identification and cross-validation of individual genes and pathways that have consistent, though maybe subtle effects on BMD variation across multiple individual uni-omics levels;2) we focused on local (cis) effects for methylation data since trans effects in epigenetic studies were generally more difficult to detect robustly. Therefore, we chose not to study it in this analysis of relatively small samples [50]; 3) we did not carry on confirmation experiments (i.e. bisulfite pyrosequencing, RT-PCR, etc) to provide stronger evidence for our findings in the present, because of the limited fund and precious limited sample in our lab. In addition, as indicated above, the replication power is usually small for initial genetic or epigenetic findings for polygenic traits; 4) the subjects are young and they are at peak bone mass. Both peak bone mass and subsequent bone loss are both important in the pathophysiology and risk of osteoporosis in the elderly such as postmenopausal women. The subjects had extremely different BMD and thus differential risk to osteoporosis, as BMD is the most powerful predictor for osteoporosis risk. It has been well and widely accepted in the field that peak bone mass is a most powerful predictor of osteoporosis risk later in life (e.g., [51–53].

In summary, through integrating the transcriptomic and epigenomic data, we identified BMD-related genes, pathways and regulatory networks which provided a much more comprehensive view of osteoporosis etiology than can be achieved by examining the individual omics data on their own, and thus lead to novel insights into the mechanisms of pathogenesis and may support the development of new therapeutics.

## Supporting Information

S1 Fig Flow cytometer analysis of the percentage of CD14^+^/CD45^+^cells from human blood. (DOCX)
