# Supplementary material for: Integrative Analysis of Transcriptomic and Epigenomic Data to Reveal Regulation Patterns for BMD Variation
Source: PLoS One. 2015 Sep 21;10(9):e0138524. doi: 10.1371/journal.pone.0138524 (PMC4577125; doi:10.1371/journal.pone.0138524)

**S1 Fig.** **Flow cytometer analysis of the percentage of CD14^+^/CD45^+^cells from human blood.** CD14 and CD45 are the specific membrane markers on monocytes and mononuclear cells, respectively.


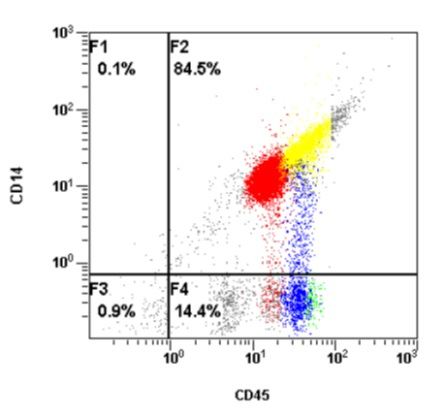

Supplement: S1 Fig — (DOCX) [file pone.0138524.s001.docx]
